# The Value of RBP4 in Assessing Coronary Artery Elasticity in Patients with Coronary Heart Disease and Type 2 Diabetes Mellitus

**DOI:** 10.31083/j.rcm2402033

**Published:** 2023-01-31

**Authors:** Yanjing Ji, Shiyu Du, Chao Tang, Jinyou Song, Xiaosong Gu

**Affiliations:** ^1^Department of Cardiology, No. 904 Hospital of Joint Logistics Support Force of PLA, 214000 Wuxi, Jiangsu, China; ^2^Department of Cardiology, the second affiliated hospital of Soochow University, 215000 Suzhou, Jiangsu, China

**Keywords:** retinol binding protein 4, coronary heart disease, type 2 diabetes mellitus, intravascular ultrasound, elasticity parameters

## Abstract

**Background:**

Existing research has shown that retinol binding protein (RBP4) has an impairing effect on arterial elasticity and induces insulin resistance, but the clinical value of RBP4 in patients with coronary heart disease (CHD) combined with type 2 diabetes mellitus (T2DM) has not been investigated. This study sought to compare the complexity of coronary artery lesions and coronary artery elasticity between patients with CHD combined with T2DM and those with CHD without T2DM, analyze the risk factors affecting coronary artery elasticity, and investigate the value of RBP4 in assessing coronary artery elasticity in patients with CHD and T2DM.

**Methods:**

A total of 130 patients with confirmed CHD were consecutively enrolled, including 38 patients with CHD combined with T2DM and 92 patients with CHD without T2DM. Basic clinical data, laboratory findings, coronary angiography and intravascular ultrasound (IVUS) imaging data, and Gensini scores and coronary artery elasticity parameters were calculated in both groups. Elasticity parameters included: stiffness parameter (β), pressure-strain elastic modulus ($E_{\text{p}}$), distensibility coefficient (DC), and compliance coefficient (CC). Multiple linear regression equations were established with elasticity parameters as dependent variables to explore the factors influencing coronary artery elasticity parameters in patients within the two groups.

**Results:**

Compared with patients in the CHD without T2DM group, patients in the CHD combined with T2DM group had higher RBP4 levels, Gensini scores, β and $E_{\text{p}}$ values, and lower DC and CC values. Linear regression analysis showed that Gensini score increased with higher β and $E_{\text{p}}$ values and decreased with higher DC and CC values. In all patients in the CHD and CHD combined with T2DM groups, RBP4 was an independent risk factor for β values after correction for confounders by multiple linear regression analysis, whereas in patients in the CHD without T2DM group, the effect of RBP4 on β values was not statistically different.

**Conclusions:**

RBP4 was an independent risk factor of coronary artery elasticity in CHD patients with T2DM and in overall CHD patients, but it did not affect coronary artery elasticity in CHD patients without T2DM.

## 1. Introduction

Coronary heart disease (CHD) is the most common type of cardiovascular diseases caused by atherosclerosis. Therefore it is of great importance to determine the risk factors and underlying mechanisms associated with CHD. Diabetes is considered to be one of the major risk factors for CHD, even after adjusting for the effects of hypertension, age, and smoking [1]. Moreover, diabetes mellitus is currently exhibiting an epidemic trend worldwide [2]. A retrospective cohort study by Booth *et al*. [3] showed that patients with type 2 diabetes mellitus (T2DM) had a 2- to 4-fold higher risk of developing CHD compared to the general population, and that the mortality rate of CHD was also increased [4]. Following percutaneous coronary intervention (PCI), the risk of stent restenosis in CHD patients with T2DM was 2.5-fold higher than those without T2DM [5].

Retinol-binding protein 4 (RBP4) is a novel adipokine secreted by adipocytes and the liver, and is significantly elevated in patients with T2DM [6]. Previous studies have confirmed that RBP4 can be involved in the development of T2DM by inducing insulin resistance and impairing islet β-cell function [7, 8]. A prospective cohort study by Liu *et al*. [9] found a close association between RBP4 and CHD. The study showed the expression of RBP4 increased in aortic atherosclerosis in both humans and mice and RBP4 tended to localize in regions rich in macrophage foam cells. This study also showed that baseline RBP4 levels remained an independent predictor for adverse cardiovascular events, even after adjustment for traditional risk factors [9].

These findings on the correlation between RBP4, T2DM and CHD suggest that elevated RBP4 levels act as an important risk factor for the progression of coronary lesions in patients with CHD combined with T2DM [10]. As an integral part of the cardiovascular system, changes in arterial elasticity often occur in the early stages of the disease and could reflect dysfunction of the entire cardiovascular system. Arterial elasticity has been shown to be an important predictor for the development of cardiovascular disease [11, 12]. There are currently several tools available for the measurement of coronary artery elasticity function. Intravascular ultrasound (IVUS), an invasive examination, is able to obtain images of lumen changes inside the coronary vessels for the complete cardiac cycle. Combined with the measurement of intracoronary pressure changes, the use of IVUS could allow more accurate calculation of elasticity parameters. In fact, this tool has already been used to measure pulmonary artery elasticity in patients with pulmonary hypertension [13].

This study sought to investigate the value of RBP4 in assessing coronary artery elasticity in patients with CHD combined with T2DM, to investigate new therapeutic strategies to improve the prognosis of these patients.

## 2. Methods

### 2.1 Patient Population

Patients with stable CHD who were admitted to the Department of Cardiology of the Second Affiliated Hospital of Soochow University from February 2017 to October 2021 and had coronary angiography and IVUS completed during their hospitalization were divided into two groups according to whether they had T2DM or not. A total of 130 patients were enrolled, including 38 patients in the CHD with T2DM group (31 males and 7 females) and 92 patients in the CHD without T2DM group (78 males and 14 females). All enrolled patients provided informed consent, and the study was approved by the Ethics Committee of the Second Affiliated Hospital of Soochow University.

CHD was defined as cardiac disease with ≥50% stenosis in at least one major coronary artery or its major branches confirmed by coronary angiography or IVUS. The diagnostic criteria of T2DM were in accordance with the ‘Standards of Medical Care in Diabetes-2021’ published by the American Diabetes Association [14]. Patients were excluded if (1) there was a ≥50% stenosis of the left main stem by coronary angiography or IVUS, and a ≥50% stenosis within 10 mm of the proximal right coronary segment. (2) A combination of severe cardiomyopathy and valvular heart disease. (3) A combination of severe hepatic and renal insufficiency, gastrointestinal bleeding, or cerebral hemorrhage. (4) A combination of acute and chronic infectious diseases, such as urinary tract infection, or biliary tract infection. (5) A combination of autoimmune diseases, such as systemic lupus erythematosus or ankylosing spondylitis. (6) A combination of malignant tumor or hematological system diseases.

Clinical information collected included age, gender, height, weight, a past medical history (history of hypertension, diabetes, atrial fibrillation, stroke, myocardial infarction, PCI, and heart failure) and a personal history (history of smoking and drinking). Fasting blood was collected from in the early morning of the day after admission and sent for RBP4, hemoglobin, serum albumin, triglycerides, total cholesterol, low-density lipoprotein cholesterol (LDL-C), high-density lipoprotein cholesterol (HDL-C), C-reactive protein (CRP), glucose, creatinine, urea nitrogen, cardiac troponin T, creatine kinase-MB (CK-MB), and N-terminal pro-B-type natriuretic peptide (NT-proBNP).

### 2.2 Imaging Data 

#### 2.2.1 Coronary Angiography

Coronary angiography was performed in all patients by interventional physicians specializing in cardiology. The determination of the degree of coronary stenosis, combined with computer-assisted quantification, was discussed and determined by at least two experienced physicians. The Gensini score was calculated according to the location of the lesion and the degree of stenosis in each coronary artery and its branches via the angiographic findings [15].

#### 2.2.2 IVUS Examination and Calculation of Elasticity Parameters

After successful completion of coronary angiography in all patients, IVUS was then performed. All IVUS images are burned to a CD after the procedure, and the results are analyzed offline (Boston Scientific Image Viewer 1.6, Boston Scientific, Marlborough, MA, USA). Changes in heart rate and pressure were monitored and recorded in real time during the IVUS examination including coronary systolic pressure (Ps), diastolic pressure (Pd) and heart rate (HR).

**Fig. 1.**
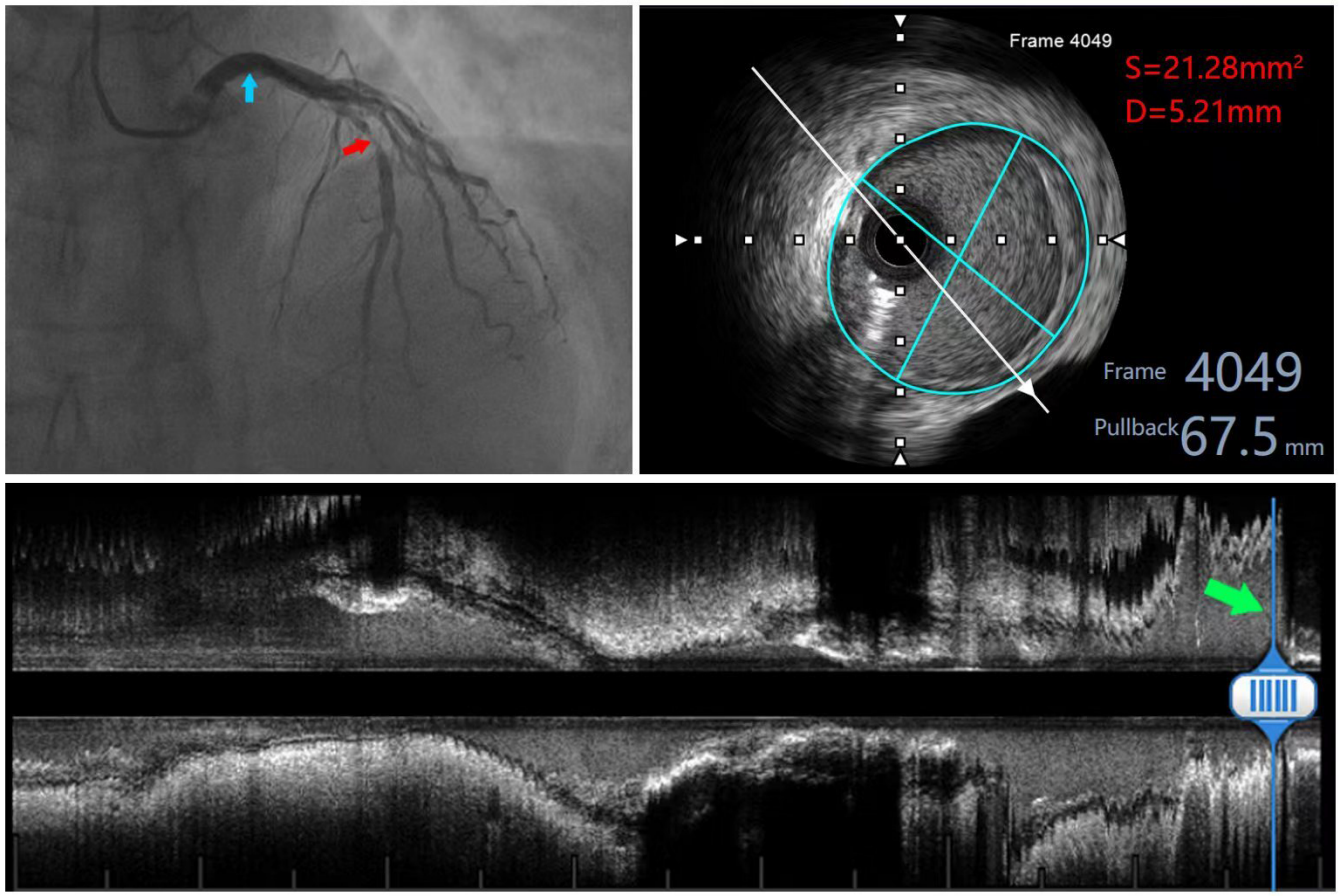
**Measurement image of a patient with left coronary artery lesion**. The red arrow in the upper left panel indicates the anterior descending branch lesion, and the blue arrow indicates the measurement site of the left main stem; the upper right panel shows the measured area and diameter within the EEM of the left main stem; the lower panel shows the long-axis image of the left coronary artery, and the green arrow indicates the measurement site.

**Fig. 2.**
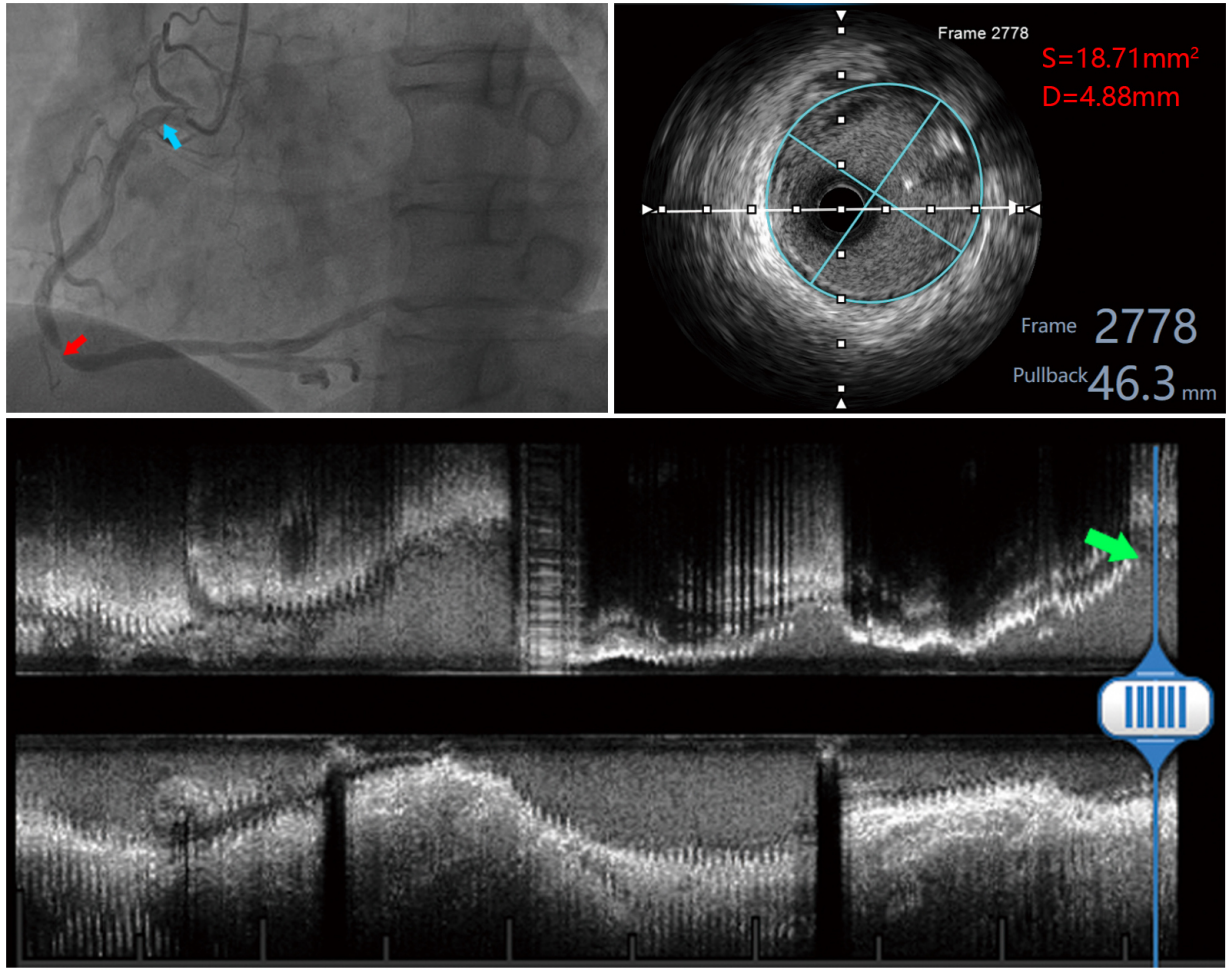
**Measured images of a patient with a right coronary artery lesion**. The red arrow in the upper left panel indicates the right coronary artery lesion, and the blue arrow indicates the measurement site proximal to the right coronary; the upper right panel shows the measured area and diameter within the EEM of the proximal right coronary; the lower panel shows the long-axis image of the right coronary artery, and the green arrow indicates the measurement site.

The offline analysis of the IVUS images was performed by two independent individuals, blinded to each other’s measurements. We evaluated inter- and intraobserver reliability for elasticity parameters, including stiffness parameter (β), pressure-strain elastic modulus (Ep), distensibility coefficient (DC), and compliance coefficient (CC) in a randomly selected sample of 50 participants. For the purposes of intraobserver reliability, all 50 IVUS images were re-analyzed (in random order) by 1 of the readers, the results were evaluated by an expert. In all 130 patients, IVUS images were measured during three consecutive cardiac cycles , and the results of the measured vessel area were averaged. When the patient had a left coronary artery lesion, the maximum vessel area (Smax) and the minimum vessel area (Smin) wrapped by the external elastic membrane (EEM) during a complete cardiac cycle were measured at the proximal end of the left main stem. The formula for the circle area was then used to find the corresponding vessel diameter, including the maximum vessel diameter (Dmax) and the minimum vessel diameter (Dmin), as shown in Fig. 1. When the patient had a right coronary lesion, the same approach was taken to obtain the area and diameter of the proximal right coronary vessels, as shown in Fig. 2. A total of four elasticity parameters were measured in this study. (1) Stiffness parameter (β) =$\frac{\ln P\,s-\ln P\,d}{\frac{\left(D\,m\,a\,x-D\,m\,i\,n\right)}{D\,m\,i\,n}}$. The larger the value of β, the smaller the change in vessel diameter and the worse the elasticity of the vessel under the same blood pressure. (2) Pressure-strain elastic modulus ($E_{\text{p}}$) =$\frac{P\,s-P\,d}{\frac{\left(D\,m\,a\,x-D\,m\,i\,n\right)}{D\,m\,i\,n}}$. $E_{\text{p}}$ is the ratio of the change in blood pressure to the relative change in the diameter of the blood vessel. The smaller the value of $E_{\text{p}}$, the better the elasticity of the blood vessels. (3) Distensibility coefficient (DC) =$\frac{\frac{\left(S\,m\,a\,x-S\,m\,i\,n\right)}{S}}{P\,s-P\,d}$. S refers to the average cross-sectional area of the blood vessel. The larger the value of DC, the better the elasticity of the vessel. (4) Compliance coefficient (CC) =$\frac{S\,m\,a\,x-S\,m\,i\,n}{P\,s-P\,d}$. The higher the value of CC, the better the vascular elasticity.

**Fig. 3.**
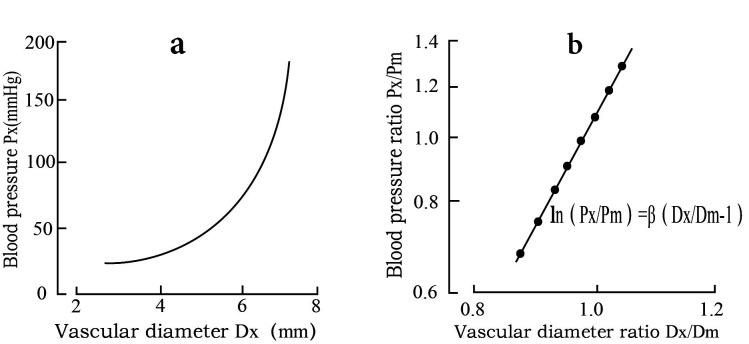
**Formulaic link between vascular diameter and blood pressure**. (a) The blood pressure-vascular diameter curve of the human artery. (b) The definition of stiffness parameter β after adjustment by mathematical equations.

Non-invasive elastic parameters are commonly used clinically to quantify arterial elastic function based on the linear correlation of blood pressure-diameter curves. However, HaYaShi *et al*. [16] found a non-linear correlation of blood pressure-diameter curves in arterial vessels after a dissection, a characteristic unique to soft biological tissues. As shown in Fig. 3a, the change in vessel diameter that can be caused by the same blood pressure gradually decreases and the vascular elasticity gets worse when blood pressure increases. This type of change makes it difficult to avoid the calculation of elasticity parameters from being influenced by fluctuations in blood pressure, even if they vary between systolic and diastolic blood pressure. HaYaShi *et al*. [16] proposed a hardness parameter to solve this problem by setting a standard pressure Pm, for example, 100 mmHg, and determining the diameter Dm at that pressure and calculating Px/Pm and Dx/Dm. When ln (Px/Pm) was further calculated and plotted against Dx/Dm, a linear relationship was seen at blood pressures in the physiological range, as shown in Fig. 3b. Since the set diameter Dm at standardized pressure was not clinically accessible, Hirai *et al*. [17] then optimized the formula to obtain the above formula (1) and confirmed that β values were not affected by fluctuations of blood pressure, while $E_{\text{p}}$ decreased in parallel with the decrease in systolic blood pressure.

The aim of our study was to study the stiffness of the coronary artery by IVUS and measure serum RBP4 levels in patients with CHD combined with or without T2DM, and to analyze the relationship between RBP4 and coronary artery stiffness.

### 2.3 Statistical Analysis

SPSS 26.0 (IBM Corp., Armonk, NY, USA) was used for statistical analysis of the collected data, and GraphPad Prism 9.0 software (Dotmatics, Boston, MA, USA) was used for graphing the statistical results. Data with normal distribution were statistically described by mean ± standard deviation, and data with non-normal distribution were described by median (quartiles).

Comparisons of continuous variables between two groups which conformed to a normal distribution with equal variance were performed using the independent sample *t*-test. The Mann-Whitney nonparametric test was used for comparison between groups that did not conform to a normal distribution. The effect of each coronary artery elasticity parameter on the Gensini score was analyzed by simple linear regression. Multiple linear regression equations were established with a β value as the dependent variable to explore the factors influencing coronary artery elasticity parameters in the total number of patients and in patients within the two groups, respectively. All tests for statistical significance were two-sided, and differences of *p *< 0.05 were considered statistically significant.

## 3. Results

### 3.1 Comparison of General Clinical Data

**Fig. 4.**
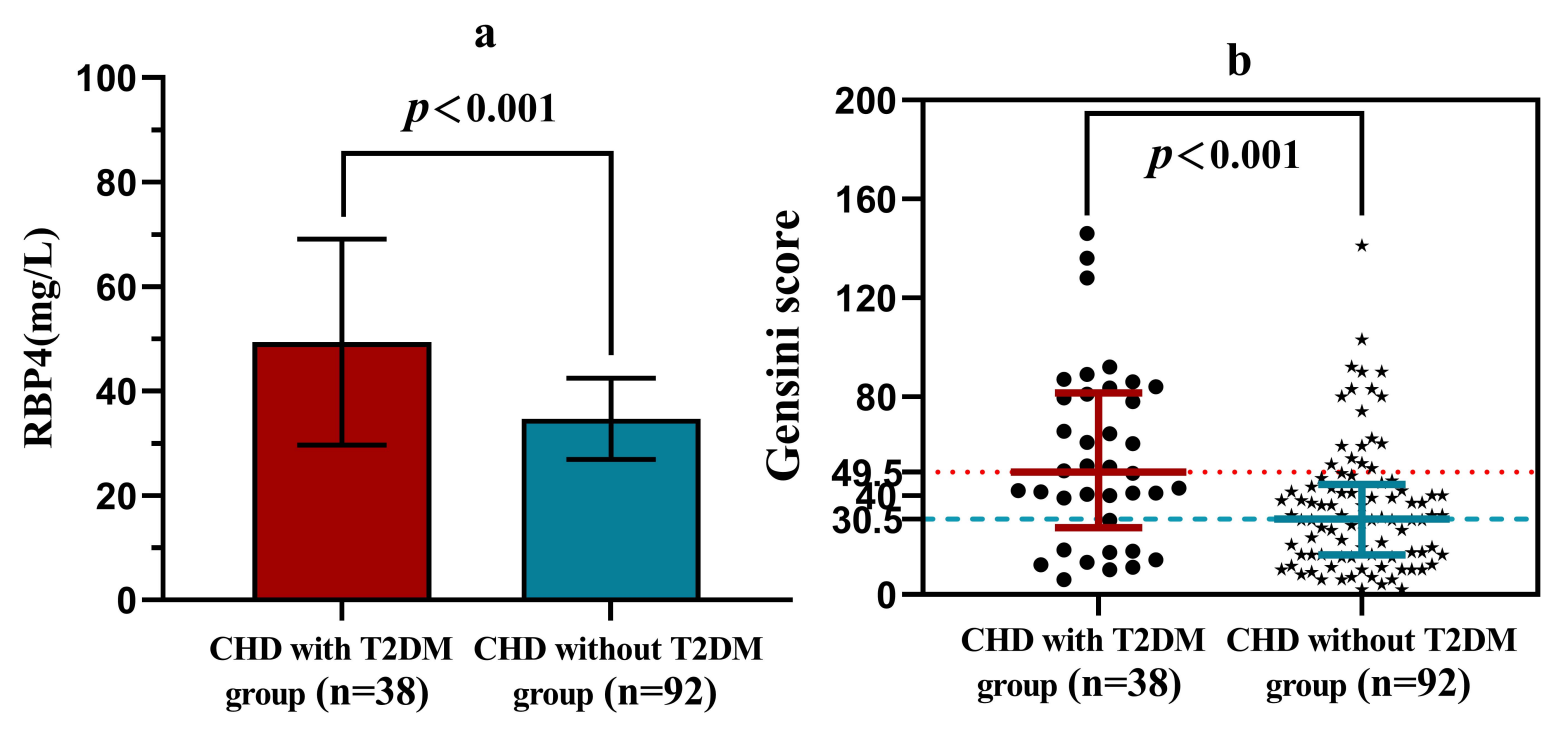
**Higher RBP4 levels and Gensini scores in patients in the CHD with T2DM group compared to the CHD without T2DM group**. (a) Comparison of RBP4 levels between the two groups of patients. (b) Comparison of the Gensini score between the two groups of patients.

Patients in the CHD with T2DM group had higher RBP4 levels compared to the CHD without T2DM group (49.26 ± 19.70 vs. 34.67 ± 7.78, *p *< 0.001), Fig. 4a. Their levels of LDL-C, triglyceride and glucose were also higher, while levels of Hb were lower. In addition, age, gender, BMI, history of smoking, history of alcohol, hypertension, stroke, atrial fibrillation, heart failure, history of myocardial infarction, history of PCI, HDL-C, total cholesterol, creatinine, urea nitrogen, albumin, CRP, CK-MB, cardiac troponin T, and NT-proBNP were not statistically different between the two groups (*p *> 0.05). The results are shown in Table 1.

**Table 1.** **Comparison of general clinical data between the two groups**.

		CHD with T2DM	CHD without T2DM	*p* value
		group (n = 38)	group (n = 92)	
Age, years		58 (49,69)	66 (48,76)	0.140
Gender (male), n (%)		31 (81.58)	78 (84.78)	0.652
BMI (kg/$m^{2}$)		25.15 ± 3.32	24.47 ± 2.30	0.256
Past medical history				
	Smoking, n (%)	19 (50.00)	42 (45.65)	0.651
	Drinking, n (%)	4 (10.53)	9 (9.78)	0.898
	Hypertension, n (%)	16 (42.11)	36 (39.13)	0.753
	Stroke, n (%)	4 (10.53)	5 (5.43)	0.447
	Atrial fibrillation, n (%)	3 (7.89)	5 (5.43)	0.691
	Heart failure, n (%)	1 (2.63)	4 (4.35)	0.645
	Myocardial infarction, n (%)	9 (23.68)	14 (15.22)	0.250
	PCI, n (%)	11 (28.95)	22 (23.91)	0.549
Laboratory assessment				
	RBP4,mg/L	49.26 ± 19.70	34.67 ± 7.78	<0.001
	LDL-C, mmol/L	2.52 (1.66, 3.90)	1.96 (1.46, 2.97)	0.049
	HDL-C, mmol/L	1.05 (0.81, 1.34)	1.10 (0.94, 1.68)	0.152
	Triglycerides, mmol/L	1.61 (1.05, 2.15)	1.25 (0.85, 1.78)	0.048
	Total cholesterol, mmol/L	3.69 ± 0.23	4.07 ± 0.13	0.122
	Hb, g/L	131.05 ± 18.31	138.56 ± 16.27	0.023
	Creatinine, umol/L	77.00 (68.00, 90.75)	77.50 (63.50, 89.00)	0.632
	Urea nitrogen, mmol/L	5.55 (4.55, 6.70)	5.60 (4.53, 6.80)	1.000
	Albumin, g/L	41.87 ± 3.92	40.45 ± 4.56	0.098
	Glucose, mmol/L	6.06 (5.32, 8.03)	5.20 (4.85, 5.77)	<0.001
	CRP, mg/L	5.40 (5.20, 5.70)	5.50 (5.10, 5.78)	0.357
	CK-MB, ng/mL	1.82 (1.29, 2.93)	1.77 (1.14, 3.30)	0.986
	Cardiac troponin T, pg/mL	12.00 (6.75, 27.75)	10.00 (4.00, 20.00)	0.125
	NT-proBNP, pg/mL	229.00 (104.75, 767.25)	164.00 (56.80, 429.75)	0.109

CHD, coronary heart disease; T2DM, type 2 diabetes mellitus; BMI, body mass index; PCI, percutaneous coronary intervention; RBP4, Retinol-binding protein 4; LDL-C, low-density lipoprotein cholesterol; HDL-C, high-density lipoprotein cholesterol; Hb, hemoglobin; CRP, C-reactive protein; CK-MB, creatine kinase-MB; NT-proBNP, N-terminal pro-B-type natriuretic peptide.

### 3.2 Comparison of Procedure-Related Information 

**Fig. 5.**
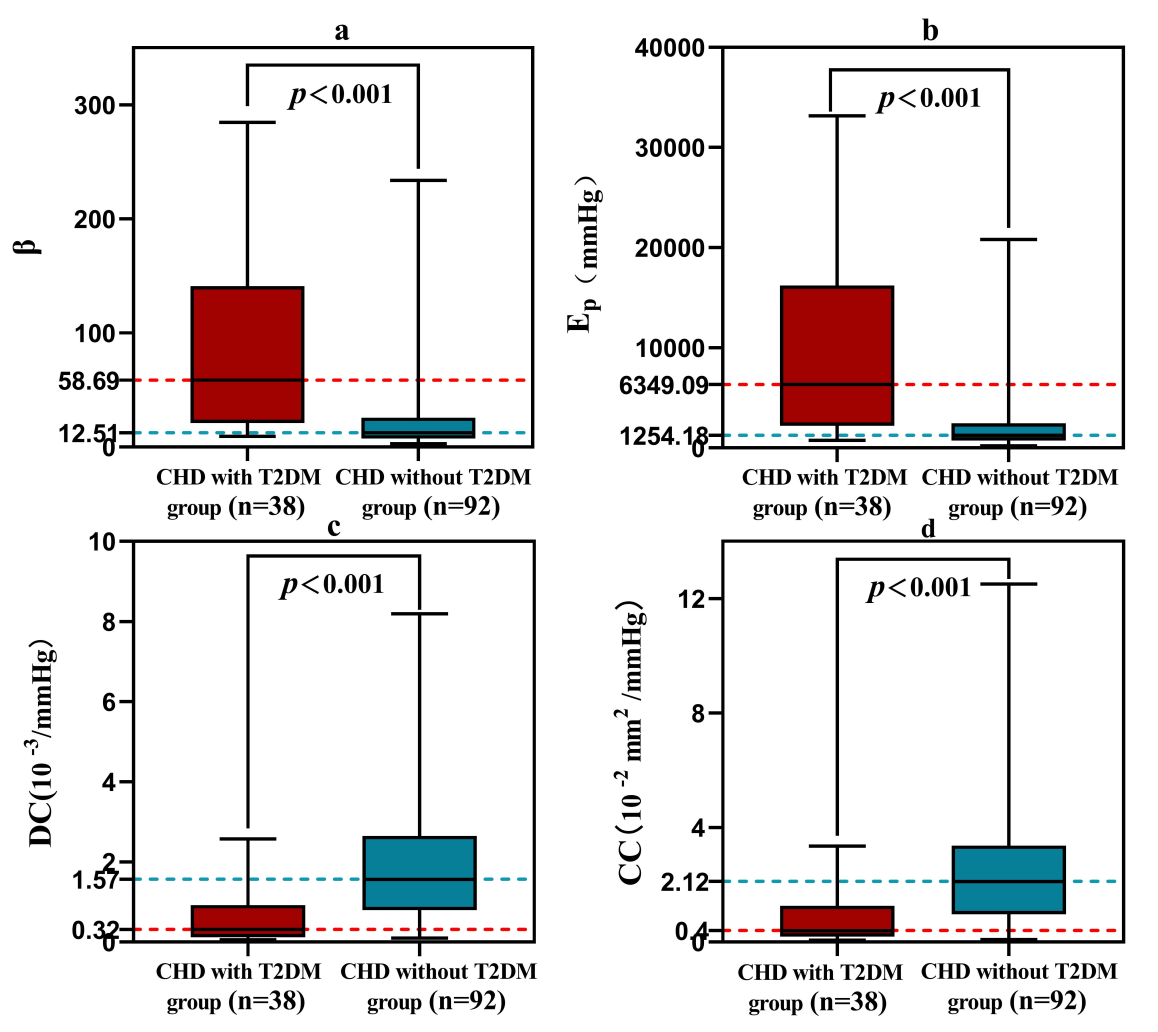
**The difference of each elasticity parameter between patients in the CHD with T2DM group and the CHD without T2DM group**. (a) Comparison of β values between the two groups of patients. (b) Comparison of $E_{\text{p}}$ values between the two groups of patients. (c) Comparison of DC values between the two groups of patients. (d) Comparison of CC values between the two groups of patients.

Compared with the CHD without T2DM group, patients in the CHD with T2DM group had a higher proportion of multivessel coronary lesions, higher Gensini scores [49.50 (27.00, 81.63) vs. 30.50 (16.00, 44.63), *p* = 0.001] (Fig. 4b), higher Ps values, higher Pd, and higher pulse pressure. In elasticity parameters, patients in the CHD with T2DM group had higher values of β [58.69 (21.08, 140.98) vs. 12.51 (7.41, 25.77), *p *< 0.001] (Fig. 5a) and $E_{\text{p}}$ [6349.09 (2215.68, 16224.85) vs. 1254.18 (729.20, 2473.61), *p *< 0.001] (Fig. 5b) than those in the CHD without T2DM group, but had lower values of DC [0.32 (0.13, 0.92) vs. 1.57 (0.80, 2.65), *p *< 0.001] (Fig. 5c) and CC [0.40 (0.19, 1.27) vs. 2.12 (0.97, 3.37), *p *< 0.001] (Fig. 5d). HR, Smax, Smin, Dmax, and Dmin were not statistically different (*p *> 0.05). The results are shown in Table 2.

**Table 2.** **Comparison of procedure-related information between the two groups**.

		CHD with T2DM	CHD without T2DM	*p* value
		Group (n = 38)	Group (n = 92)	
Multiple vascular lesions, (%)		23 (60.53)	37 (40.22)	0.035
Gensini score		49.50 (27.00, 81.63)	30.50 (16.00, 44.63)	0.001
Ps, mmHg		137.95 ± 14.92	125.60 ± 13.94	<0.001
Pd, mmHg		76.61 ± 10.24	71.89 ± 9.78	0.015
Pulse pressure, mmHg		61.34 ± 10.62	53.71 ± 10.55	<0.001
HR, times/min		75.21 ± 12.56	73.22 ± 10.99	0.369
Smax, ${\mathrm{mm}}^{2}$		14.15 (10.79, 17.71)	13.22 (10.87, 16.99)	0.620
Smin, ${\mathrm{mm}}^{2}$		13.82 (10.25, 16.92)	12.52 (9.86, 14.50)	0.122
Dmax, mm		4.25 (3.71, 4.75)	4.10 (3.72, 4.66)	0.612
Dmin, mm		4.20 (3.61, 4.64)	3.99 (3.55, 4.30)	0.123
Elasticity parameters				
	β	58.69 (21.08, 140.98)	12.51 (7.41, 25.77)	<0.001
	$E_{\text{p}}$, mmHg	6349.09 (2215.68, 16224.85)	1254.18 (729.20, 2473.61)	<0.001
	DC, ${\mathrm{10}}^{-3}$/mmHg	0.32 (0.13, 0.92)	1.57 (0.80, 2.65)	<0.001
	CC, ${\mathrm{10}}^{-2}$ ${\mathrm{mm}}^{2}$/mmHg	0.40 (0.19, 1.27)	2.12 (0.97, 3.37)	<0.001

CHD, coronary heart disease; T2DM, type 2 diabetes mellitus; Ps, systolic blood pressure; Pd, diastolic blood pressure; HR, heart rate; Smax; maximum vessel area; Smin, minimum vessel area; Dmax, maximum vessel diameter; Dmin, minimum vessel diameter; β, stiffness parameter; $E_{\text{p}}$, pressure-strain elastic modulus; DC, distensibility coefficient; CC, compliance coefficient.

### 3.3 Simple Linear Regression Analysis of Gensini Score and Each Elasticity Parameter

**Fig. 6.**
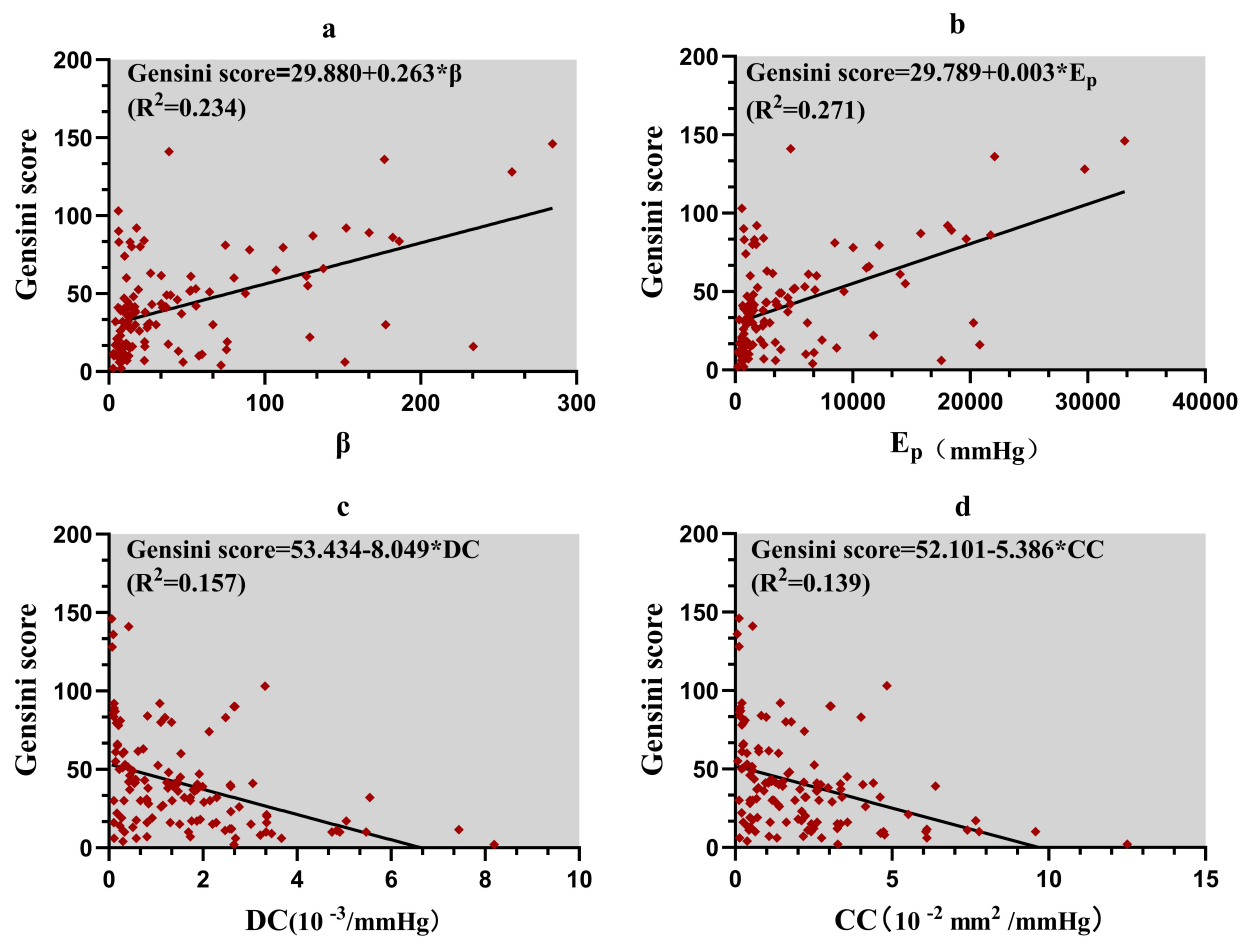
**The linear correlation between Gensini score and each elasticity parameter**. (a) Effect of β values on the Gensini score. (b) Effect of $E_{\text{p}}$ values on the Gensini score. (c) Effect of DC values on the Gensini score. (d) Effect of CC values on the Gensini score.

The scatter plot shows a linear correlation between Gensini score and each elasticity parameter. To further analyze the effect of coronary artery elasticity on the severity of coronary lesions in patients with CHD, simple linear regression analysis was performed with Gensini score as the dependent variable and elastic parameters β, $E_{\text{p}}$, DC, and CC as independent variables, respectively. The results showed that the effect of each elasticity parameter on the Gensini score were statistically significant , as shown in Table 3 and Fig. 6.

**Table 3.** **Simple linear regression analysis of Gensini score and each elasticity parameter**.

Variables	Coefficient	Standard deviation	Standardization coefficient	*p* value	95% Confidence interval (CI)
β	0.263	0.042	0.484	<0.001	0.180–0.346
$E_{\text{p}}$	0.003	0.0004	0.521	<0.001	0.002–0.003
DC	–8.049	1.647	–0.397	<0.001	(–11.310)–(–4.792)
CC	–5.386	1.184	–0.373	<0.001	(–7.729)–(–3.045)

β, stiffness parameter; $E_{\text{p}}$, pressure-strain elastic modulus; DC, distensibility coefficient; CC, compliance coefficient.

### 3.4 Analysis of the Influencing Factors of Elasticity Parameters

The advantages of β over other elasticity parameters were demonstrated through the calculation of elasticity parameters in the previous section. In order to reduce the influence of blood pressure differences on the authenticity of coronary artery elasticity parameters, β was chosen as the dependent variable to establish multiple linear regression analysis. To explore the risk factors affecting coronary artery elasticity, single-factor linear regression analyses were first performed, and then independent variables with *p *< 0.2 were screened out for stepwise multiple linear regression analyses.

#### 3.4.1 Multiple Linear Regression Analysis for All CHD Patients

Univariate linear regression analysis was established in all CHD patients with β values as the dependent variable with age, gender, BMI, history of smoking and drinking, hypertension, atrial fibrillation, heart failure, LDL-C, HDL-C, triglyceride, total cholesterol, Hb, glucose, RBP4, CRP, CK-MB, cardiac troponin T, NT-proBNP, and T2DM as independent variables, respectively. The results screened out the following variables as influencing factors for β values—BMI (*p* = 0.007), RBP4 (*p *< 0.001), age (*p *< 0.001), creatinine (*p* = 0.013), LDL-C (*p *< 0.001), HDL-C (*p *< 0.001), hypertension (*p *< 0.001), smoking history (*p *< 0.001), T2DM (*p *< 0.001). Then, the above variables were incorporated into the multiple linear regression model, and the following variables were found to significantly affect β values—RBP4 (*p *< 0.001), hypertension (*p *< 0.001), LDL-C (*p *< 0.001), age (*p *< 0.001), and T2DM (*p *< 0.001), as detailed in Table 4.

**Table 4.** **Multiple linear regression analysis for all CHD patients**.

Variables	Univariate analysis			Multivariate analysis		
	Coefficient (95% CI)	Standardization coefficient	*p* value	Coefficient (95% CI)	Standardization coefficient	*p* value
RBP4	2.96 (2.505–3.414)	0.751	<0.001	1.330 (0.909–1.751)	0.338	<0.001
Hypertension	64.205 (47.977–80.433)	0.569	<0.001	31.451 (20.629–42.273)	0.279	<0.001
LDL-C	28.223 (20.627–35.818)	0.545	<0.001	10.401 (5.761–15.041)	0.201	<0.001
Age	2.414 (1.890–2.938)	0.627	<0.001	0.878 (0.487–1.268)	0.228	<0.001
T2DM	61.450 (43.111–79.789)	0.506	<0.001	33.371 (22.354–44.387)	0.275	<0.001
BMI	4.916 (1.341–8.490)	0.234	0.007			
Creatinine	0.215 (0.046–0.384)	0.217	0.013			
HDL-C	–51.665 ((–69.401)–(–33.928))	–0.454	<0.001			
Smoking	59.055 (42.666–75.444)	0.533	<0.001			

RBP4, Retinol-binding protein 4; LDL-C, low-density lipoprotein cholesterol; T2DM, type 2 diabetes mellitus; BMI, body mass index; HDL-C, high-density lipoprotein cholesterol.

#### 3.4.2 Multiple Linear Regression Analysis for Patients in the CHD with T2DM Group

Univariate regression analysis was established with β values of patients in the CHD with T2DM group as the dependent variable, with age, gender, BMI, history of smoking and drinking, hypertension, atrial fibrillation, heart failure, LDL-C, HDL-C, triglyceride, total cholesterol, Hb, glucose, RBP4, CRP, CK-MB, cardiac troponin T, and NT-proBNP as independent variables, respectively. The results screened out the following variables as influencing factors for β values—BMI (*p* = 0.031), RBP4 (*p *< 0.001), age (*p *< 0.001), LDL-C (*p *< 0.001), HDL-C (*p *< 0.001), hypertension (*p *< 0.001), and smoking history (*p *< 0.001). The above variables were incorporated into the multiple linear regression model, and the following variables were found to significantly affect β values—RBP4 (*p* = 0.031), hypertension (*p* = 0.045), LDL-C (*p* = 0.001), and age (*p* = 0.033), as shown in Table 5.

**Table 5.** **Multiple linear regression analysis for patients in the CHD with T2DM group**.

Variables	Univariate analysis			Multivariate analysis		
	Coefficient (95% CI)	Standardization coefficient	*p* value	Coefficient (95% CI)	Standardization coefficient	*p* value
RBP4	3.323 (2.751–3.894)	0.891	<0.001	1.185 (0.118–2.252)	0.318	0.031
Hypertension	120.009 (91.417–148.600)	0.817	<0.001	34.304 (0.835–67.773)	0.234	0.045
LDL-C	43.319 (29.306–57.332)	0.722	<0.001	16.602 (7.297–25.907)	0.277	0.001
Age	3.65 (2.879–4.421)	0.848	<0.001	1.066 (0.092–2.039)	0.248	0.033
BMI	7.785 (0.772–14.798)	0.351	0.031			
HDL-C	–101.302 ((–141.552)–(–61.051))	–0.648	<0.001			
Smoking	111.854 (80.675–143.032)	0.772	<0.001			

RBP4, Retinol-binding protein 4; LDL-C, low-density lipoprotein cholesterol; BMI, body mass index; HDL-C, high-density lipoprotein cholesterol.

#### 3.4.3 Multiple Linear Regression Analysis for Patients in the CHD Without T2DM Group

Univariate regression analysis was established with β values in patients in the CHD without T2DM group as the dependent variable, while with age, gender, BMI, history of smoking and drinking, hypertension, atrial fibrillation, heart failure, LDL-C, HDL-C, triglyceride, total cholesterol, Hb, glucose, RBP4, CRP, CK-MB, cardiac troponin T, and NT-proBNP as independent variables, respectively. The following variables were screened out as influencing factors for β values—RBP4 (*p* = 0.104), age (*p *< 0.001), LDL-C (*p* = 0.001), HDL-C (*p *< 0.001), hypertension (*p *< 0.001), and smoking history (*p *< 0.001). The above variables were then incorporated into the multiple linear regression model, and the following variables were still found to significantly affected β values—hypertension (*p *< 0.001), LDL-C (*p* = 0.001), and age (*p* = 0.002), as detailed in Table 6.

**Table 6.** **Multiple linear regression analysis for patients in the CHD without T2DM group**.

Variables	Univariate analysis			Multivariate analysis		
	Coefficient (95% CI)	Standardization coefficient	*p* value	Coefficient (95% CI)	Standardization coefficient	*p* value
Hypertension	38.442 (27.158–49.726)	0.581	<0.001	28.273 (16.948–39.599)	0.427	<0.001
LDL-C	11.718 (5.206–18.231)	0.353	0.001	9.047 (3.942–14.153)	0.272	0.001
Age	1.257 (0.812–1.703)	0.509	<0.001	0.683 (0.259–1.108)	0.277	0.002
RBP4	0.713 (–0.148–1.574)	0.171	0.104			
HDL-C	–24.139 ((–37.019)–(–11.260))	–0.365	<0.001			
Smoking	34.065 (22.508–45.622)	0.525	<0.001			

LDL-C, low-density lipoprotein cholesterol; RBP4, Retinol-binding protein 4; HDL-C, high-density lipoprotein cholesterol.

## 4. Discussion

T2DM has been found to influence and participate in the development of CHD. Its effect on vascular endothelial cells and vascular elasticity is particularly significant and has important pathological significance. Endothelial dysfunction is one of the pathological bases of CHD [18]. Quagliaro *et al*. [19] found that the concentration of superoxide anion radical (${\mathrm{O2}}^{-}$) increases during periods of hyperglycemia. This results in oxidative damage, enhances the expression of cysteinyl aspartate specific proteinase-3 (caspase-3), inhibits the expression of B-cell lymphoma-2 (Bcl-2), which has anti-apoptotic effects, and thus induces endothelial cell apoptosis. Another study found that the ratio of tetrahydrobiopterin (BH4) to dihydrobiopterin (BH2) was reduced in patients with T2DM [20]. In addition, BH2 reduces the bioavailability of BH4 by competitively binding to endothelial-type nitric oxide synthase (eNOS), a key cofactor of eNOS. This action reduces endothelial-derived NO production, which is a key factor in the regulation of vascular tone and endothelial function. Furthermore, Yoshida *et al*. [21] found that hyperglycemia-induced accumulation of advanced glycated end products (AGEs) in the vessel wall or glycosylation of the vascular extracellular matrix significantly reduces vascular compliance and increases the incidence of CHD in patients with T2DM.

This study found that in addition to T2DM; RBP4, age, hypertension and LDL-C significantly affected coronary artery elasticity, which is generally consistent with the results of previous studies [22, 23, 24, 25]. A study by Chondrou *et al*. [22] regarding the effect of RBP4 on arterial elasticity, found that RBP4 was significantly associated with aortic stiffness after adjusting for the influence of age and pulse pressure, as RBP4 levels increased, arterial elasticity gradually decreased. Aging is also a known factor contributing to the decrease of the elastic function of large arteries. The effect of age on arterial elasticity is partly attributed to changes in the extracellular matrix within the arterial wall, such as degradation of elastin fibers and increased formation of cross-linked molecules such as AGEs [23]. In addition, ageing-induced imbalance in the vasoactive molecular environment, vascular oxidative stress and chronic inflammation are also thought to reduce arterial compliance. The negative effects of hypertension on vascular elastic function have also been demonstrated in clinical practice. Sustained high circulatory load in patients with hypertension will damage the elastin structure of the arterial wall, causing remodeling of the vessel wall and lumen enlargement, as well as inducing phenotypic changes in arterial endothelial and smooth muscle cells to decrease arterial elasticity [24]. Finally, Chen *et al*. [25] found in a cross-sectional study, that the risk of atherosclerosis increased with increasing duration of LDL-C exposure in a dose-dependent manner, demonstrating that elevated LDL-C levels will further decrease arterial compliance.

In this study, in order to determine whether the effect of RBP4 on coronary artery elasticity was different in patients with CHD with and without T2DM, a multiple linear regression analysis was established by using the β value as the dependent variable in both groups. The results showed that RBP4 could significantly affect the coronary artery elasticity of patients in the CHD with T2DM group but not affect that of patients in the CHD without T2DM group. The potential mechanism of the effect of RBP4 on coronary artery elasticity will require further investigation.

The effect of RBP4 on arterial elasticity may be partly attributed to its induction of insulin resistance in endothelial cells and the reduction of endothelium-derived NO production [26]. The mechanisms of insulin action on arterial endothelial cells have now been extensively elucidated [27, 28]. After binding to endothelial cell surface receptors, insulin can increase endothelial-derived NO production through the PI3K/Akt signaling pathway, and act on the mitogen-activated protein kinase (MAPK) signaling pathway to promote the secretion of endothelin-1 (ET-1), which has a strong vasoconstrictive effect and promotes the expression of adhesion factors. Insulin resistance (IR) could selectively inhibit the PI3K/Akt-eNOS-NO signaling pathway without reducing ET-1 secretion, and the imbalance between the two would impair arterial vasodilatory function [28].

RBP4 can also reduce arterial compliance and accelerate atherosclerosis progression by impairing mitochondrial function and inducing endothelial apoptosis in arterial endothelial cells. The PI3K/Akt signaling pathway not only is involved in regulating endothelial-derived NO production, but also functions to regulate the activity of the Bcl-2 family proteins [29]. An increase in the Bax/Bcl-2 ratio leads to a change in mitochondrial membrane permeability, increasing the release of mitochondrial cytochrome C (Cyt C) and promoting apoptotic events, whereas a decrease in the Bax/Bcl-2 ratio has the opposite effect. Wang *et al*. [10] found that RBP4 increased mitochondrial reactive oxygen species (ROS) production in human aortic endothelial cells (HAECs) in a dose-dependent manner, and decreased mitochondrial content, integrity and membrane potential. In either RBP4-treated HAECs or the transgenic mice expressing human RBP4 (RBP4-Tg), Cyt C expression and Bax/Bcl-2 ratio were found to be significantly elevated, which would eventually lead to apoptotic events in arterial endothelial cells. Combined with changes in phosphorylation levels at specific downstream sites (Ser473, Thr308), this suggests that high levels of RBP4 will inhibit the PI3K/Akt signaling pathway and induce apoptosis in aortic endothelial cells.

RBP4 may affect coronary artery elasticity by promoting the proliferation and migration of vascular smooth muscle cells (VSMCs), which exhibit a fully functional and differentiated phenotype under physiological conditions and express contractile proteins important for maintaining vascular tone. However, even though VSMCs are highly differentiated and mature, they are still highly plastic and can undergo a phenotypic transition from “contractile” to “synthetic” phenotype under conditions of injury or induction [30]. The phenotypic transition from a “contractile” to a “synthetic” phenotype is characterized by a decrease in myofilament density and contractile protein expression, which are replaced by an increase in the expression of proinflammatory factors and extracellular matrix [31]. For example, VSMCs can be involved in vascular calcification by transforming into osteoblasts. Zhou *et al*. [32, 33] found that RBP4 promoted the development of atherosclerosis in both diabetic rats by regulating the JAK2/STAT3 signaling pathway and rat aortic smooth muscle cells in a high-glucose environment, further validating that high levels of RBP4 promoted the proliferation of VSMCs by regulating this pathway. This further verified that high levels of RBP4 promoted the proliferation and migration of VSMCs and the occurrence of diabetic macrovascular events through the regulation of this pathway, consistent with the view in our study that RBP4 is a risk factor for the progression of coronary artery lesions in CHD patients with T2DM.

Finally, The relationship between pericardial fat thickness and coronary heart disease has been closely studied in recent years. Epicardial adipose tissue (EAT) volume (EAV) can be used to diagnose high-risk coronary plaque burden associated with coronary events [34]. Further research shows that pericoronary adipose tissue is closely related to atherosclerotic plaque formation. Right coronary artery Pericoronary adipose tissue computed tomography attenuation(PCATa) has prognostic value beyond clinical characteristics [35]. Several studies have found that EAV has a negative relationship with artery stiffness [36, 37]. The mechanism by which coronary peripheral fat or pericardial peripheral fat increases the risk of coronary atherosclerosis remains unclear. Salgado-Somoza *et al*. [38] showed that Retinol-binding protein 4 is expressed in EAT and subcutaneous adipose tissue (SAT), and that RBP4 protein levels were higher in EAT from CAD than non-CAD patients. Therefore, RBP4 produced by EAT is another risk factor for the progression of coronary artery lesions in CHD. Since RBP4 is produced by adipose tissue, one of the reasons for the increase of RBP4 in CHD patients may be due to the high proportion of adipose tissue or BMI in these patients. Controlling body fat to reduce the level of RBP4 may be an important measure to reduce the occurrence of coronary heart disease in diabetic patients.

Our patients all had diabetes and coronary heart disease and received medication to control blood sugar, hypertension and lipid levels. Weather these medical therapies can influence RBP4 levels is still unclear. These medications and dietary modifications will impact levels of oxidative stress and lifestyle changes such as cessation of smoking could modulate RBP4 concentrations [39]. Fortunately, both our study groups had similar clinical characteristics and these factors had limited impact on our results. In CHD patients, plaque component and plaque elasticity had a significant positive relationship with artery stiffness [40, 41]. Therefore, the left main coronary artery and the proximal part of the right coronary artery without plaque were selected as the locations for measuring arterial elasticity.

Our study is innovative in that the obtained coronary elasticity parameters were analyzed in correlation with RBP4 by using IVU. Furthermore, this study reveals that the adipokine RBP4 is an independent risk factor for coronary artery elasticity and shows differences in levels between CHD patients with and without T2DM, providing a new therapeutic strategy for patients with CHD combined with T2DM. However, this study still has some limitations. First, this study was a cross-sectional study and did not demonstrate the time-dependent effect of RBP4 on coronary artery elastic function. Second, the small sample size was small and derived from a single-center in Suzhou. Third, this study was based on the population with stable CHD, so the results cannot be extended to acute patients. Fourth, our study did not show RBP4 was an independent risk factor for vascular stiffness in patients with CHD without T2DM. It could be that the number of patients without diabetes and with significant vascular stiffness was too low. Finally, the correlation between RBP4, arterial stiffness and clinical outcomes had not been investigated, so further clinical studies are needed to understand whether reducing RBP4 levels in diabetic patients with CHD may actually have an impact on prognosis. This topic will need further investigation future studies.

## 5. Conclusions

Our study found that RBP4 was an independent risk factor for coronary artery elasticity in patients with CHD combined with T2DM and in all CHD patients, but it did not affect the coronary artery elasticity of CHD patients without T2DM. This suggests that RBP4 is important for the assessment of coronary artery elasticity in patients with CHD combined with T2DM and that treatment targeting RBP4 may decelerate the progression of coronary artery lesions in these patients.

## Data Availability

The data and materials that support the findings of this study are openly available from the corresponding author upon reasonable resquest.

## Abbreviations

β, stiffness parameter; Ep, pressure-strain elastic modulus; DC, distensibility coefficient; CC, compliance coefficient; AGEs, advanced glycated end products; EEM, external elastic membrane; caspase-3, cysteinyl aspartate specific proteinase-3; Bcl-2, B-cell lymphoma-2; BH2, dihydrobiopterin; BH4, tetrahydrobiopterin; eNOS, endothelial nitric oxide synthases; MAPK, mitogen-activated protein kinase; ET-1, endothelin-1; HAECs, human aortic endothelial cells; Bax, BCL2-associated X protein; VSMCs, vascular smooth muscle cells.
